# Neural correlates of emotional working memory predict depression and anxiety

**DOI:** 10.3389/fnins.2025.1574901

**Published:** 2025-05-14

**Authors:** Leiting Li, Meirong Sun, Mengdi Qi, Yiwen Li, Dongwei Li

**Affiliations:** ^1^Department of Psychology, Beijing Sport University, Beijing, China; ^2^Department of Psychology, Faculty of Arts and Sciences, Beijing Normal University, Zhuhai, China; ^3^Laboratory of Sports Stress and Adaptation of General Administration of Sport, Beijing Sport University, Beijing, China; ^4^Key Laboratory of Exercise and Physical Fitness (Beijing Sport University), Ministry of Education, Beijing, China; ^5^Experimental Teaching Platform, Beijing Normal University, Zhuhai, China; ^6^State Key Laboratory of Cognitive Neuroscience and Learning & IDG/McGovern Institute for Brain Research, Beijing Normal University, Beijing, China; ^7^Beijing Key Laboratory of Applied Experimental Psychology, National Demonstration Center for Experimental Psychology Education, Faculty of Psychology, Beijing Normal University, Beijing, China

**Keywords:** emotional deficit, working memory, EEG, transdiagnostic methods, anxiety and depression

## Abstract

**Introduction:**

Emotional working memory (WM) plays a critical role in cognitive functions such as emotion regulation, decision-making, and learning. Understanding how emotional stimuli, particularly negative ones, affect WM performance is crucial for identifying cognitive markers of mental health issues like anxiety and depression. Our objective is to determine whether trait anxiety and depression levels are associated with specific performance outcomes in emotional WM and whether behavioral and neural indicators demonstrate statistically significant correlations with individual anxiety and depression levels in university students.

**Methods:**

In our research: Experiment 1 (*n* = 25) tested WM performance with both positive and negative emotional stimuli under different cognitive loads (2 vs. 4 items), while Experiment 2 (*n* = 34) combined EEG recording to investigate the neural index of anxiety and depression during negative emotional WM.

**Results:**

Results showed that negative emotional stimuli impaired WM performance, especially under higher cognitive loads, with anxiety level being linked to increased theta activity during encoding and depression level associated with decreased alpha activity during retrieval. Additionally, individuals with higher anxiety exhibited reduced sensitivity to cognitive load differences in WM tasks involving negative emotions.

**Discussion:**

These results demonstrated that specific EEG patterns during negative emotional WM were significantly associated with individual anxiety and depression levels, suggesting the potential utility of EEG measures for identifying at-risk individuals of anxiety and depression in university student populations. By linking cognitive and neural indicators, the study contributes to the development of personalized interventions for mental health monitoring and treatment.

## Introduction

Working memory (WM) is an essential cognitive system believed to underlie many higher-order cognitive functions, including learning, reasoning, which also plays a crucial role in emotion regulation (Baddeley, 2012; Levens and Gotlib, 2010). WM involves three cognitive processes: encoding, maintenance, and retrieval. Each representing distinct cognitive operations of information in WM and arising from separate neural sources (Hale et al., 1996; Bledowski et al., 2006a,b; Pinal et al., 2014).

Emotional WM is defined as the ability to effectively hold emotional contexts in WM (Schweizer et al., 2013; Barker and Bialystok, 2019), which imposes greater cognitive demands (Schweizer et al., 2013; Janus and Bialystok, 2018; Bai et al., 2019; Katie et al., 2019). The significance of emotional WM is particularly evident in education, for example, to assess whether students can resist emotional interference and manage emotional information processing effectively (Janus and Bialystok, 2018). In education contexts, emotional WM serves as tools for evaluating emotion regulation abilities and predicting real-life problem-solving skills and social adaptation competencies (Ladouceur et al., 2009; Schweizer et al., 2013; Engen and Anderson, 2018; Janus and Bialystok, 2018).

Previous studies have investigated the effects of emotional valence in image material on WM updating task, emotional stimuli can influence WM updating task performance due to the emotions they elicit. Some studies utilized emotional valence in images to induce affective states in participants, followed by tasks requiring memorization of neutral content (e.g., letters or colors; Xie and Zhang, 2016; Huang et al., 2021; Plancher et al., 2019). In contrast, some researches focused on the direct memorization of emotionally valenced content, Rozovskaya et al. (2016) examined whether emotional valence influences participants’ sensitivity to perceptual details in images, while Levens and Gotlib (2010) assessed the ability to continually update emotion representations in WM by requiring participants to identify facial emotions.

Mather et al. (2006) found that high-arousal negative images impaired spatial WM for their locations compared to low-arousal images. Similarly, two related studies examined emotional interference in WM and found high-arousal negative emotional stimuli disrupted visual WM more than positive stimuli (Iordan and Dolcos, 2017; García-Pacios et al., 2015). A recent study found performance differences in a 2-back task that required matching the hue (blue or yellow) or valence (negative or neutral) of emotional images (Hur et al., 2017). They observed slower performance in the N-back task when images were negative, suggesting that negative emotional materials might impair WM. Contrarily, Jackson et al. (2009) found that negative faces enhanced visual WM performance compared to happy and neutral faces. Gotoh et al. (2010) reported similar effects with textual materials, suggesting that negative affective valence may enhance WM performance.

Despite the growth in behavioral studies on WM in emotional disorders (Schweizer et al., 2019), the conclusions are still under debate. This may be due to the difference in the way emotional stimuli are presented (related to or unrelated to the task, different ways of induction), and the difference in the setting of the control group (compared with neutral emotions or directly compared with positive and negative emotions), which makes it difficult to compare and form unity between different studies. In addition, the facilitative effect of emotional stimuli on the performance of emotional WM tasks may occur at specific stages of WM. A recent study revealed that the enhanced representations of emotional stimulus were found during the encoding and maintenance stages of WM, but not during retrieval (Jackson et al., 2014). Similarly, there is evidence that positive and negative emotional stimulus facilitate the resolution of interference during WM (Levens and Phelps, 2008). However, the neurophysiological underpinnings of encoding, maintenance, and retrieval processes of emotional WM in students not diagnosed as emotional disorder remain poorly understood.

In the current study, to investigate how individual differences in anxiety and depression modulate performance in emotional WM tasks, we instructed participants to memorize emotional content. When selecting stimuli from standardized databases, we ensured balanced complexity and content categories (e.g., human, animals, scene) across negative and positive valences. Additionally, to mitigate potential interference from repeated exposure to identical stimuli, the encoding and retrieval phases within each trial of the emotional WM task were segregated.

In electrophysiological research, theta activity (4—7 Hz) has been closely associated with the control of information held in WM (Mitchell et al., 2008; Hsieh and Ranganath, 2013). Increased theta activities during WM tasks have been consistently observed across different experimental paradigms (Gevins, 1997; Jensen and Tesche, 2002) and sensory modalities (Bastiaansen et al., 2002; Sauseng et al., 2007). A frequently reported finding is the concurrent increase in theta activity with WM loads (Jensen and Tesche, 2002; Onton et al., 2005; Sauseng et al., 2007). Moreover, a positive relationship between theta activity and behavioral performance has been documented in multiple studies (Gevins, 1997; Smith et al., 1999; Itthipuripat et al., 2013). Some studies have recently utilized task-related frequency band measures to investigate how negative emotions such as anxiety and depression impact performance in WM tasks. Research has confirmed that induced stress conditions leading to decreased task performance are associated with reduced theta activity (Gaertner et al., 2014). On the other hand, recent research has identified oscillations in the alpha band (8–13 Hz) as a neural correlate of attention during WM processing (Figueira et al., 2020). Selective attention enhances the encoding of goal-relevant representations while preventing the storage of irrelevant or distracting information (Chadick and Gazzaley, 2011; Vogel et al., 2005). It is believed that changes in alpha power are inversely related to cortical excitability; specifically, an increase in alpha power is associated with cortical idling, whereas a decrease in alpha power correlates with heightened excitability in the visual cortex (Pfurtscheller et al., 1996). Although no recognition differences were found between emotional and neutral representations in the emotional WM tasks, it has been observed that increased alpha power may reflect a shift in attentional focus from emotional representations to processes requiring greater attention demands (Macedo-Pascual et al., 2019). Therefore, our study aims to distinguish groups with varying levels of anxiety and depression using task-related frequency band measures.

Negative emotions inherent to an individual’s traits, such as depression and anxiety, have been shown to adversely affect general WM capacity. For example, clinical depression patients exhibit impairments in both visual and verbal WM capacity (Christopher and MacDonald, 2005), and individuals with high math anxiety may show deficits in verbal WM (Ashcraft and Kirk, 2001; Elliman et al., 1997). Similarly, a study involving individuals with social anxiety, found that higher levels of anxiety symptoms were associated with poorer verbal WM performance (Waechter et al., 2018). Importantly, the decline in WM capacity across different cognitive loads in WM tasks was significantly correlated with the severity of anxiety and depression. Recent research has demonstrated that WM can regulate emotions through manipulation of cognitive loads (Ja and Pa, 2019). For instance, a recent study observed that, comparing to healthy controls, individuals with anxiety disorders exhibited impaired behavioral performances only in 3-back, but not in 1-back (Vytal et al., 2016). In contrast, Van Dillen and colleagues found that as the cognitive loads of WM tasks increased, the intensity of self-reported negative emotional responses decreased (Van Dillen et al., 2009; Van Dillen and Koole, 2007). Therefore, although the direction remains unclear, the variable of cognitive load may play a crucial role in moderating the relationship between negative emotional states such as anxiety and depression, and performance on WM tasks. We proposed employing continuous variables to assess participants’ levels of anxiety and depression, instead of simply dichotomizing them, to explore more nuanced variations which were taken by manipulating cognitive load.

Importantly, the comorbidity rate between depression and anxiety has been estimated to reach as high as 60% for case-level disorders (Kessler et al., 2015), with the rate likely being even higher when including subthreshold cases. Previous research suggests that a transdiagnostic approach may offer greater utility in conceptualizing these disorders (Brand, 2015). Our study adopts a transdiagnostic approach to assess anxiety and depression as continuous variables. By utilizing this approach, we can isolate the comorbid elements of anxiety and depression, thereby identifying the unique factors associated with each disorder. This methodological refinement enables a more precise evaluation of the independent effects of anxiety and depression on emotional WM.

Although the impact of trait anxiety and depression on working memory has been observed in clinical populations (Amir and Bomyea, 2011; Levens and Gotlib, 2010), how trait anxiety and depression, as continuous variables, affect emotional WM task performance in university students who were not diagnosed as emotional disorder remain unclear. A recent meta-analysis demonstrated a strong association between generally high anxiety and lower working memory capacity, with this association being weaker in subclinical populations compared to clinical populations (Moran, 2016). However, the boundary for categorizing individuals as trait anxious or non-anxious in different studies have not been consistent. In contrast, studying trait anxiety and depression as continuous variables in the general population is more reasonable. Following transdiagnostic methods for anxiety and depression among clinical populations, we examined trait anxiety and depression levels in the general population, extracting specific factors associated with anxiety and depression. The extent to which trait anxiety and depression are associated with emotional WM performance, and whether related EEG patterns show consistent correlations with these trait measures, requires further investigation.

Our study aims to investigate the neural mechanisms underlying emotional WM and the relationship between individual anxiety/depression and emotional WM performance. Experiment 1 compares the impact of positive and negative emotional stimuli on WM task performance under different cognitive loads. Experiment 2 explores the influence of individual trait anxiety and depression levels on the performance of a negative emotional WM task and on neural activities based on time-domain electroencephalogram (EEG) signals. Our objective is to examine (a) the association between trait anxiety/depression levels and specific performance measures in emotional working memory tasks, and (b) the potential relationships between behavioral/neural indicators and individual differences in anxiety and depression level.

## Methods

### Participants

Using G*Power 3.1 software (Faul et al., 2007), the required sample size for the study was calculated. For Experiment 1 (a 2*2 repeated measures ANOVA), with an effect size of 0.5, an alpha level of 0.05, and a desired power of 0.80, the minimum required sample size was determined to be 20 participants. For Experiment 2 (a paired-samples *t*-test), under the same parameters, the required sample size was calculated to be 34 participants. Based on these results, 25 students (ages: 18–24 years, mean age 21.04 years, 16 females) participated in the experiment 1 and 35 students (ages: 18–24 years, mean age 20.37 years, 17 females) participated in the experiment 2. All participants from Beijing Sport University were right-handed and had normal or corrected-to-normal vision. Written informed consent was obtained from all participants in accordance with the Declaration of Helsinki. Data from 1 participant from experiment 2 were excluded for further analysis because of low EEG quality. Therefore, EEG data from 34 participants (ages: 18−24 years, mean age 20.35 years, 17 females) were included in the final analysis of experiment 2 (Table 1). The study protocol was approved by the local Ethics Committee.

**Table 1:** Demographic information (M ± SD).

Measures	Experiment 1 (*n* = 25)	Experiment 2 (*n* = 34)
Age (year)	21.04 ± 2.15	20.35 ± 1.52
Depression level	4.88 ± 2.68	4.71 ± 4.13
Anxiety level	4.4 ± 2.90	3.82 ± 4.02

M, mean; SD, standard deviation.

### Questionnaire measurement

**Figure 1:**
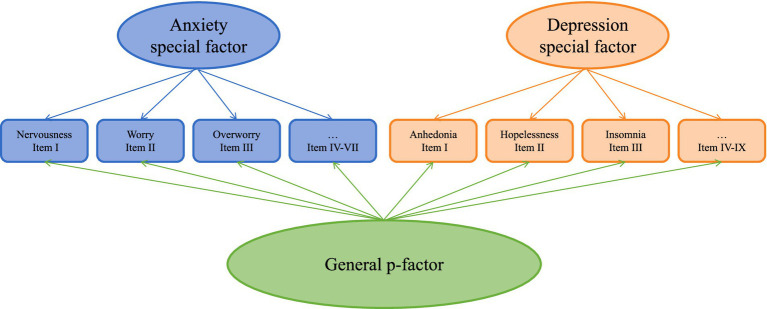
Bifactor model structure of anxiety and depression symptoms. The diagram illustrates a bifactor model with one general psychopathology factor (p-factor) and two orthogonal specific factors (Anxiety and Depression). Anxiety-specific factor indicators: I. Nervousness, II. Worry, III. Overworry, IV. Tension, V. Restlessness, VI. Irritability, VII. Apprehension (GAD-7 items). Depression-specific factor indicators: I. Anhedonia, II. Hopelessness, III. Insomnia, IV. Fatigue, V. Appetite changes, VI. Guilt, VII. Concentration difficulties, VIII. Psychomotor symptoms, IX. Suicidal ideation (PHQ-9 items). All items additionally load on the general p-factor (not shown for clarity). Roman numerals correspond to original questionnaire item numbers. Model constraints include: (a) no cross-loadings between anxiety items and depression factor (or vice versa), and (b) factor variances fixed to 1 for identification.

Participants completed a series of questionnaires before the formal experiment, which were primarily used for evaluating dimensions of emotion and some personality characteristics. The Patient Healthy Questionnaire (PHQ-9; Kroenke et al., 2001) is a 10-item scale which assesses the severity of depression. General Anxiety Disorder-7 (GAD-7; Spitzer et al., 2006) is a 7-item scale assessing the presence of a clinically significant anxiety disorder. We used the Bifactor Model specifying a general p-factor and two orthogonal specific factors for anxiety and depression, which has been evident that is the best model for the comorbidity structure of anxiety and depression (Lee et al., 2024; Brodbeck et al., 2011; Simms et al., 2008). This model includes 7 symptoms of anxiety and 9 symptoms of depression (Figure 1). Each item was specified to load simultaneously on a general p-factor and a specific factor (anxiety or depression), with cross-loadings constrained to zero to ensure structural clarity. To achieve model identification, the variances of the latent factors (p-factor, anxiety, and depression) were fixed to 1. We implemented this bifactor structure in Mplus using robust maximum likelihood estimation (MLR), which employs a sandwich estimator to adjust standard errors for potential non-normality and non-independence of observations. The MLR method further accounts for non-normal data distributions without requiring transformations.

### Tasks

**Figure 2:**
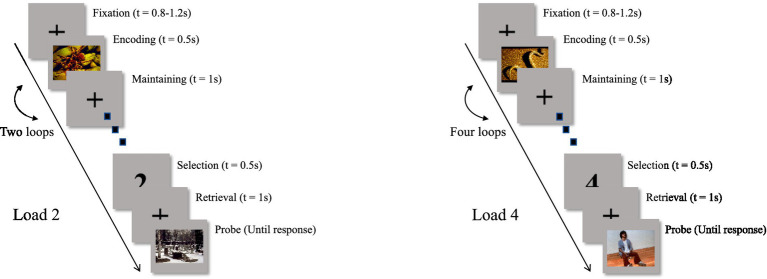
Emotional WM task. Trial sequences of the emotional WM task with Load 2 and Load 4.

In experiment 1, participants completed the sequential emotion WM task (Figure 2), which employed a two-factor experimental design (WM loads: Load 2 vs. Load 4; emotional stimuli: positive vs. negative). The task consisted of evenly distributed trials/conditions, with a total of 6 blocks, each load containing 30 trials, resulting in 180 trials in total. The rest period between two blocks was determined by the participants. Participants were seated approximately 60 cm from the screen. In each trial, a central fixation point was presented for 0.8–1.2 s. During the encoding phase, an emotional image was randomly presented at the center of the screen for 0.5 s. The images (10.16° height × 15.36° width), were selected from a set of 18 images with validated positive or negative emotional valence, with 9 images in each category (negative: valence *M* = 3.45, *SD* = 0.59, arousal *M* = 4.70, *SD* = 0.54; positive: valence *M* = 6.71, *SD* = 0.90, arousal *M* = 5.32, *SD* = 0.35). All images are from The International Affective Picture System (IAPS; Bradley and Lang, 2007), which has been widely used (Rozovskaya et al., 2016; Xie and Zhang, 2016; Hendricks and Buchanan, 2016). Following the encoding phase, a fixation appeared for 1 s during the maintaining phase. Each cycle consisted of one encoding and one maintaining phase. Trials with Load 2 involved two cycles, while those with Load 4 involved four cycles. After the cycles, a number was randomly presented during the selection phase: either 1 or 2 in Load 2 trials, or 1 to 4 in Load 4 trials. This number indicated which image from the respective sequence the participants needed to recall. Subsequently, a one-second fixation was shown during the retrieval phase. In the probe phase, an image from the encoding phase’s source was randomly presented, and participants had to determine whether it matched the image recalled during the selection phase and respond accordingly. After the participants made their judgment, the trial concluded and the next trial began. The experiment concluded after completing all 6 blocks and 180 trials. The sole difference in task between experiment 1 and experiment 2 is that experiment 2 only has negative emotional materials. This was done to obtain enough trials to improve the signal-to-noise ratio of EEG data, mainly focusing on the relationship between negative emotional WM and depression and anxiety.

### Transparency and openness

We report all data exclusions, manipulations, and measures used in this study. The study design was not preregistered. All stimulus materials, raw data, and analysis scripts are available on the Open Science Framework.^1^

### EEG recording and preprocessing

In experiment 2, sixty-four channels Neuroscan (SynAmps2, NuAmps) and online acquisition software Curry7 were used for EEG recording, simultaneously recorded vertical and horizontal EOG (electrooculogram). CPz served as reference electrode during recording. EEG channels were named according to the closest corresponding channel in the 10–20 system (Chatrian et al., 1985). The signals were sampled at a rate of 1,000 Hz. The electrode resistance was reduced to less than 10 kΩ before beginning the formal experiment.

EEG preprocessing was conducted using custom Matlab scripts and the EEGLAB (Delorme and Makeig, 2004). EEG data were resampled at 500 Hz, then re-referenced to the average of the two mastoids and band-pass filtered between 0.1 Hz and 40 Hz. Additionally, a 48–52 Hz band-pass filter was applied to remove 50 Hz power line noise. The potential influence of EMG (electromyographic) activity in the EEG signal was minimized by using the available EEGLAB routines (Delorme and Makeig, 2004). Independent component analysis (ICA) was implemented to detect and remove artifactual EEG components, particularly those reflecting ocular activity (Hoffmann and Falkenstein, 2008), using a semi-automated approach that combined ICLabel classification with visual inspection. Across all 34 participants, an average of 6.2 ± 1.9 independent components (range: 2–11) were rejected from 62 components per dataset. Subsequently, the EEG signal was downsampled to 500 Hz. Segments was chosen from −500 ms to 1,500 ms of the onset of the encoding and selection phase. The moving window peak-to-peak function was used for artifact removal (voltage values exceeding ±100 μV). On average, 0.68% ± 2.80% (0–12.8%) of epochs per participant were rejected, leaving 88.9 trials for Load 2 and 90.0 trials for Load 4 for further analysis.

### Time frequency analysis

After preprocessing, epochs were used for calculating time-frequency representations. Instantaneous power and phase were estimated within the 2–30 Hz frequency range using short-time Fourier transforms with Hanning taper data and a 0.5 Hz step. Power values were then normalized to the baseline period from –500 ms to 0 ms of the encoding and retro-cue onset, respectively. Event-related spectral perturbation (ERSP) was calculated for each epoch relative to the baseline period across all electrodes and converted to decibels by multiplying the log ratio by 10 (Grandchamp and Delorme, 2011). In the time-frequency plots, power reductions relative to baseline are shown in blue, while power increases are shown in yellow. Alpha (8–13 Hz) and theta (4–7 Hz) power were extracted by averaging time-frequency representations in corresponding frequency band from posterior electrodes (According to the situation of the topographies, we finally selected the following electrodes for analysis: P3/4, PO3/4, P5/6, PO5/6, P7/8, PO7/8, O1/2).

### Statistical analysis

In experiment 1, our behavioral data analysis focused on the impact of emotional stimuli and WM loads on task performance. Therefore, we calculated the accuracy and reaction time of participants under different WM loads and emotional stimuli, and conducted paired-sample *t*-tests for each condition. The behavioral data analysis in experiment 2 concentrated on the impact of WM loads on performance in the negative emotional WM task, so we calculated the accuracy and reaction time of participants under different memory loads and conducted paired-sample *t*-tests.

To elucidate the relationship between behavioral performance and individual characteristics, and to determine whether anxiety and depression affect performance on the emotional WM task, we conducted correlation analyses on selected behavioral and questionnaire data. Firstly, we calculated whether individual performance under different WM loads and emotional stimuli was significantly correlated with specific questionnaire metrics. Secondly, we assessed whether the difference in individual performance between different memory loads (under the premise that there is a significant and consistent directional difference in individual performance between different working memory loads) was significantly correlated with levels of anxiety and depression. This step aimed to investigate whether individuals with varying levels of anxiety and depression exhibit differential performance on specific emotional WM tasks.

Building on experiment 1, experiment 2 aimed to identify neural indicators associated with individual levels of anxiety and depression in task-related EEG. We conducted correlation analyses on selected behavioral, EEG and questionnaire data. First, we calculated whether individual levels of anxiety and depression were associated with certain EEG metrics. Second, we calculated whether differences in specific EEG metrics between different memory loads were significantly correlated with anxiety and depression levels. Lastly, we calculated whether high and low emotional subgroups (depression/anxiety) have different performance significantly in specific EEG metrics.

## Results

### Experiment 1

**Figure 3:**
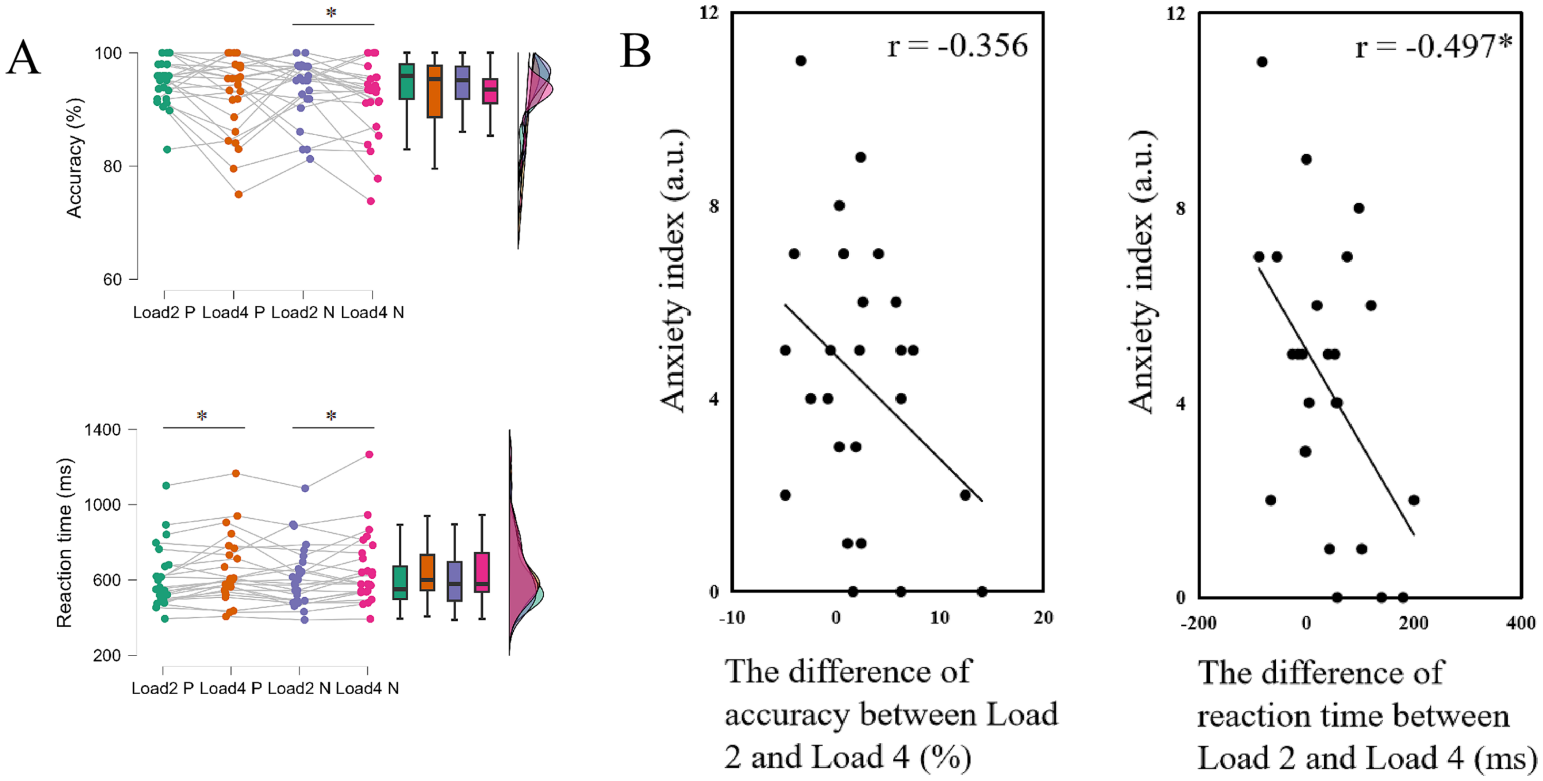
The results of experiment 1. **(A)** Behavioral outcomes of accuracy and reaction time for Load 2 positive trials, Load 2 negative trials, Load 4 positive trials and Load 4 negative trials. **(B)** Correlation between the difference in individual performance on negative emotional WM between different memory loads and anxiety index, ∗ *p* < 0.05. Load 2 P indicates the positive stimulus in Load 2 condition; Load 2 N indicates the negative stimulus in Load 2 condition (Load 4 P and Load 4 N indicate in a similar way).

To examine the effects of load and stimulus type on behavioral performance, a repeated measures ANOVA was conducted. The means and standard deviations for accuracy and reaction time under each condition are presented in Table 2. Significant main effects of load were found on both accuracy (*F*(1,24) = 7.841, *p* = 0.010, $\eta_{p}^{2}$ = 0.246) and reaction time (*F*(1,24) = 9.337, *p* = 0.005, $\eta_{p}^{2}$ = 0.280), indicating that behavioral performance declined as memory load increased (Figure 3A). The main effect of stimulus type was not significant on neither accuracy (*F*(1,24) = 1.690, *p* = 0.206, $\eta_{p}^{2}$ = 0.066) nor reaction time (*F*(1, 24) = 0.866, *p* = 0.361, $\eta_{p}^{2}$ = 0.035), suggesting that emotion might not significantly affect memory performance. No significant interaction was found (accuracy: *F*(1,24) = 0.001, *p* = 0.980, $\eta_{p}^{2}$ < 0.001; reaction time: *F*(1,24) = 0.142, *p* = 0.710, $\eta_{p}^{2}$ = 0.006).

**Table 2:** Behavioral results (M ± SD).

Measures	Load 2 P	Load 2 N	Load 4 P	Load 4 N
Accuracy (%)	94.79 ± 3.97	93.84 ± 5.34	92.53 ± 6.89	91.55 ± 6.58
Reaction time (ms)	608.82 ± 162.43	618.47 ± 166.28	649.81 ± 175.86	654.46 ± 187.06

Behavioral outcomes of accuracy and reaction time for Load 2 positive trials, Load 2 negative trials, Load 4 positive trials and Load 4 negative trials. M, mean; SD, standard deviation.

To elucidate the relationship between behavioral performance and individual characteristics, we conducted correlation analyses on selected behavioral and questionnaire data to determine whether anxiety and depression affect performance on the emotional WM task. Results showed that individuals with higher the anxiety scores exhibited smaller differences in the reaction time on negative emotional WM between different loads (accuracy: *r* = −0.356, *p* = 0.081; rt.: *r* = −0.497, *p* = 0.011; Figure 3B). These correlations were absent on positive stimulus (accuracy: *r* = 0.015, *p* = 0.943; rt.: *r* = −0.348, *p* = 0.088). These results suggested that individuals with higher anxiety levels are more sensitive to negative emotional stimuli, leading to smaller performance differences between different working memory loads. Therefore, in experiment 2, we focused on negative emotional WM and include EEG recordings to further explore the underlying neural indicators.

### Experiment 2 behavioral results

**Figure 4:**
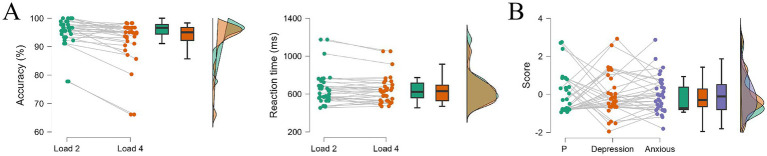
The results of experiment 2. **(A)** Behavioral outcomes of accuracy and reaction time for Load 2 trials and Load 4 trials. **(B)** Participants’ performance on P factor, depression special factor and anxiety special factor, which are transformed by depression and anxious scales, ∗ *p* < 0.05.

Figure 4A showed the accuracy and reaction time for negative emotional WM under Load 2 and Load 4 in EEG experiment, and the depression and anxiety of the participants from Experiment 2 was shown in Figure 4B. The accuracy for Load 2 and Load 4 was 95.20 ± 5.06% and 92.49 ± 7.74% respectively, and the reaction times were 659.00 ± 174.00 ms and 641.78 ± 144.94 ms, respectively. Consistent with the behavioral results in Experiment 1, we found a significant decline on accuracy from Load 2 to Load 4 (*t* = 3.869, *p* < 0.001, Cohen’s d = 0.664). However, no significant difference was found on reaction time (*t* = 1.708, *p* = 0.097, Cohen’s d = 0.293).

### Experiment 2 time-frequency representations during encoding and maintaining

**Figure 5:**
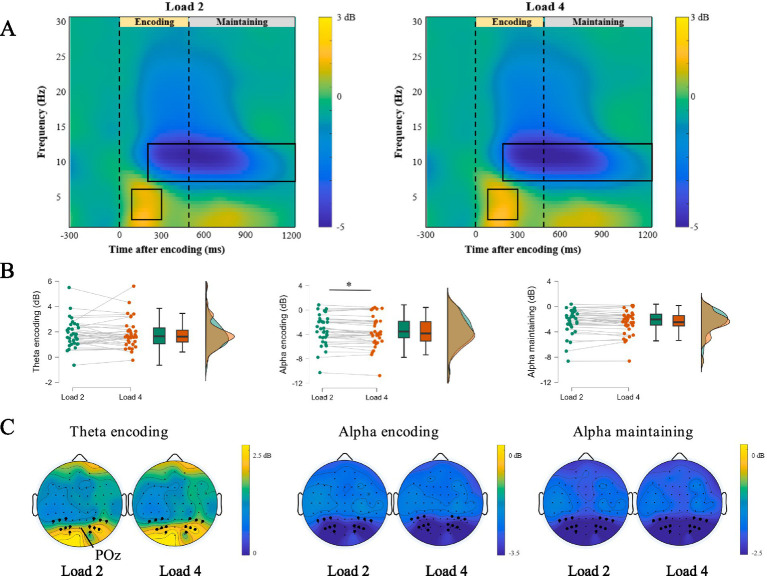
ERSP activity in encoding and maintaining phase. **(A)** The difference in the time-frequency representations of Load 2 and Load 4 workload. **(B)** The difference in the decibel of alpha and theta band in load-2 and load-4 workload, ∗ *p* < 0.05. **(C)** EEG topography in Load 2 and Load 4 trials during encoding and maintaining of negative emotional working memory task.

We first focused on the time-frequency representations during the encoding and maintaining phases of negative WM. Figures 5A,C showed time-frequency representations and corresponding topographic maps. A significant positive cluster appeared in the theta band (4–7 Hz) between 100 and 300 ms after stimulus onset (Load 2: *t* = 9.552, *p* < 0.001, Cohen’s d = 1.638; Load 4: *t* = 9.290, *p* < 0.001, Cohen’s d = 1.593), and two significant negative clusters appeared in the alpha band (8–13 Hz) between 200 and 500 ms (Load 2: *t* = −7.962, *p* < 0.001, Cohen’s d = −1.366; Load 4: *t* = −8.356, *p* < 0.001, Cohen’s d = −1.433) or between 500 and 1,200 ms (Load 2: *t* = −7.279, *p* < 0.001, Cohen’s d = −1.248; Load 4: *t* = −8.154, *p* < 0.001, Cohen’s d = −1.398).

To examine how WM loads (Load 2 vs. Load 4) affected performance in the negative emotional working memory task (Figure 5B), we calculated ERD and ERS for the relevant frequency bands. During the encoding phase (200–500 ms), the decibel values for the alpha band were −3.36 dB for Load 2 and −3.59 dB for Load 4. Paired-sample *t*-tests showed that ERD was significantly higher for Load 4 than for Load 2 (*t* = 2.56, *p* = 0.015, Cohen’s d = 0.438). However, we did not find significant differences between the Load 2 and Load 4 in the theta band of the encoding phase (Load 2: 1.81 dB, Load 4: 1.86 dB, *t* = −0.344, *p* = 0.733, Cohen’s d = −0.059) and the alpha band of the maintaining phase (Load 2: −2.45 dB, Load 4: −2.61 dB, *t* = 1.704, *p* = 0.098, Cohen’s d = 0.292). These results suggested the alpha band in the encoding phase is more sensitive to WM loads.

### Experiment 2 time-frequency representations during selection and retrieval

**Figure 6:**
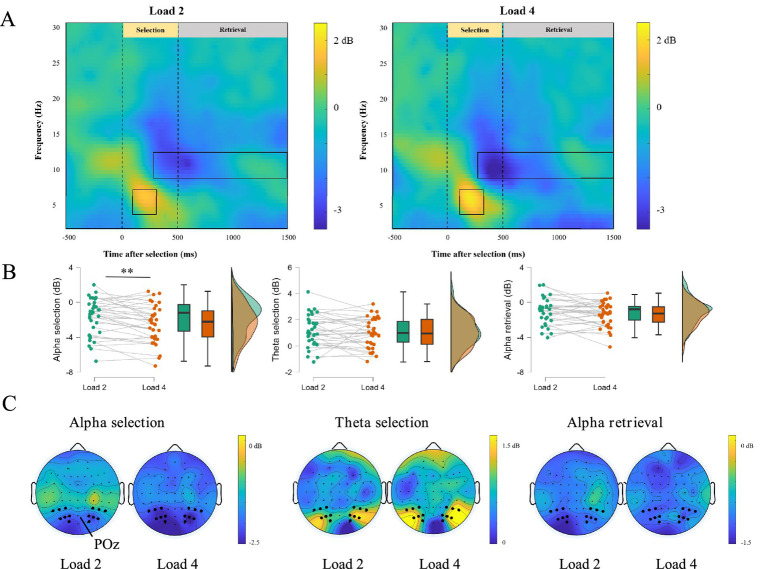
ERSP activity in selection and retrieval phase. **(A)** The difference in the time-frequency representations of Load 2 and Load 4 workload. **(B)** The difference in the decibel of alpha and theta band in Load 2 and Load 4 workload, ∗∗*p* < 0.01. **(C)** EEG topography in Load 2 and Load 4 trials during selection and retrieval of negative emotional working memory task.

ERSP activities were clearly observed during the selection and retrieval phases. Figures 6A,C showed time-frequency representations and corresponding topographic maps. A significant positive cluster appeared in the theta band (4–7 Hz) between 100–300 ms after selection onset (Load 2: *t* = 5.495, *p* < 0.001, Cohen’s d = 0.942; Load 4: *t* = 5.057, *p* < 0.001, Cohen’s d = 0.867), and a significant negative cluster appeared in the alpha band (8–13 Hz) between 200–500 ms (Load 2: *t* = −4.709, *p* < 0.001, Cohen’s d = −0.808; Load 4: *t* = −6.633, *p* < 0.001, Cohen’s d = −1.138) or between 500–1,500 ms (Load 2: *t* = −4.195, *p* < 0.001, Cohen’s d = −0.719; Load 4: *t* = −6.029, *p* < 0.001, Cohen’s d = −1.034).

We also calculated ERD and ERS for the relevant frequency bands (Figure 6B). During the selection phase (300–500 ms), the decibel values for the alpha band were −1.68 dB for Load 2 and −2.44 dB for Load 4. Paired-sample *t*-tests indicated that ERD was significantly stronger for Load 4 than for Load 2 (*t* = 3.33, *p* = 0.002, Cohen’s d = 0.572). However, we did not find significant differences between the Load 2 and Load 4 in the theta band of the selection phase (Load 2: 1.13 dB, Load 4: 0.98 dB, *t* = 0.839, *p* = 0.408, Cohen’s d = 0.144) and the alpha band of the retrieval phase (Load 2: −1.07 dB, Load 4: −1.42 dB, *t* = 1.688, *p* = 0.101, Cohen’s d = 0.290). These results suggested the alpha band in the selection phase is more sensitive to WM loads.

### Experiment 2 correlations between EEG index and questionnaires

**Figure 7:**
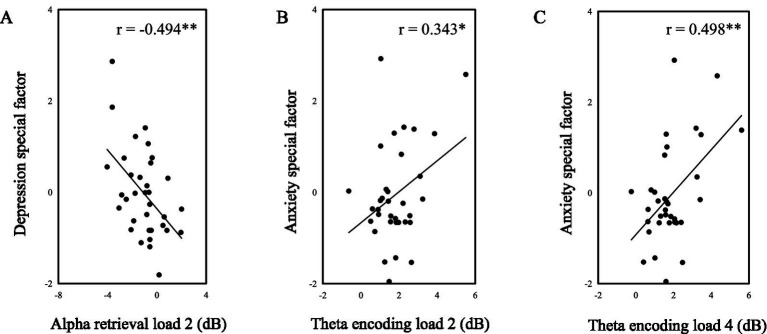
The correlation between emotion and EEG index. ∗ *p* < 0.05, ∗∗ *p* < 0.01.

Using transdiagnostic methods, we derived depression-specific and anxiety-specific factors as continuous variables and found that they were associated with certain frequency bands during specific stages of the negative emotional working memory task (Figure 7). Note that both depression-specific and anxiety-specific factors were obtained by controlling common parts of depression and anxiety. The depression-specific factor was negatively correlated with the alpha power during the retrieval phase of Load 2 (*r* = −0.494, *p* = 0.003). The anxiety-specific factor was positively correlated with the theta power during the encoding phase of both Load 2 (*r* = 0.343, *p* = 0.047) and Load 4 (*r* = 0.498, *p* = 0.003). These findings indicated that individuals with higher levels of depression had lower alpha power during the selection phase of Load 2, while individuals with higher levels of anxiety had higher theta power during the encoding phase of both Load 2 and Load 4 trials, which may reflect distinct electrophysiological patterns associated with depression and anxiety from the transdiagnostic view.

### Experiment 2 control analysis from the traditional diagnostic method

To further support the correlation results from the transdiagnostic view, we divided our data into high and low depression/anxiety subgroups. Consistent with our correlation findings, alpha power during retrieval at Load 2 showed a significant difference between high and low depression subgroups (*t* = 2.200, *p* = 0.035), and theta power during encoding also showed significant differences between high and low depression subgroups (Load 2: *t* = 1.968, *p* = 0.058; Load 4: *t* = 2.201, *p* = 0.035).

## Discussion

The current study examined electrophysiological patterns during emotional WM and their associations with depression and anxiety level among university students. The results revealed significant relationships between individual differences in anxiety and depression and specific neural and behavioral indicators during emotional WM tasks. Specifically, we found that individuals with higher levels of anxiety and depression exhibited distinct patterns in both behavioral performance and EEG signals, which underscores the complex interplay between emotional states and cognitive functions. Our research offers new insights into the use of EEG as a non-invasive and cost-effective tool for assessing neural activity, which offers a promising approach for early detection and monitoring of anxiety and depression. This has significant application value in campus mental health screening, the measure of continuous change in symptoms of depression and anxiety, and subsequent intervention target determination. By integrating neural and behavioral data, clinicians can gain a comprehensive understanding of an individual’s cognitive and emotional functioning, leading to more personalized and effective treatment strategies. In the field of education, supplementing traditional psychological health questionnaires with targeted EEG cognitive paradigm assessments for students with high scores in depression and anxiety can more effectively and accurately screen potential high-risk groups for depression and anxiety.

Previous studies have shown significantly prolonged response times and lower accuracy when processing negative emotional stimuli compared to neutral or positive counterparts (Rozovskaya et al., 2016; Xie et al., 2023). Our findings further revealed that emotional stimuli significantly impacted WM performance with the increasing cognitive loads. Participants showed varying accuracy and reaction times, depending on the emotional valence of the stimuli and the cognitive load. Notably, negative emotional stimuli under higher cognitive loads resulted in poorer performance, suggesting that negative emotions might exacerbate cognitive demands, thereby impairing WM. This aligns with prior studies indicating that negative emotions can hinder cognitive performance by increasing cognitive loads and attentional demands (Mather et al., 2006; Hur et al., 2017). Moreover, previous research showed that anxiety can lead to impaired cognitive flexibility and increased susceptibility to emotional interference (Waechter et al., 2018), while individuals with depression exhibit slowed disengagement from sad stimuli and accelerated disengagement from happy stimuli compared to non-depressed controls, reflecting maladaptive and protective working memory biases that may impede affective regulation (Levens and Gotlib, 2010). Our correlation analyses revealed a linear relationship between anxiety levels and performance in negative emotional WM tasks, higher levels of anxiety were associated with smaller performance differences between different memory loads for negative emotional tasks. This suggests that individuals with higher anxiety may have a diminished capacity to adjust their cognitive resources in response to varying task demands, potentially due to heightened sensitivity to negative emotional WM.

The EEG data provided deeper insights into the neural mechanisms underlying emotional WM. Time-frequency representations revealed significant differences between different cognitive loads. Specifically, during the encoding and retrieval phases, individuals with higher anxiety and depression levels exhibited distinct patterns in the theta and alpha bands, respectively.

During the encoding phase, the significant positive cluster observed in the theta band between 100 and 300 ms after stimulus onset suggests that encoding emotional stimuli involves substantial cognitive effort, particularly in individuals with higher anxiety. This finding suggests that individuals with higher anxiety may require greater neural effort to encode emotional information, which is reflected in increased theta activity. Theta oscillations are associated with cognitive control and the integration of emotional information (Cavanagh and Frank, 2014). This heightened theta activity indicates that anxious individuals may be more sensitive to emotional stimuli, requiring greater neural resources to process and encode emotional stimuli effectively. Previous studies have also linked increased theta activity with heightened emotional arousal and cognitive demand (Mitchell et al., 2008).

Depressed individuals did not show a similar increase in theta activity during the encoding phase. Instead, their neural response was characterized by reduced alpha activity during the retrieval phase. Alpha oscillations are typically associated with inhibitory control and the suppression of irrelevant information (Akil et al., 2024; Hwang et al., 2014). Lower alpha activity in individuals with higher depression suggests a potential deficit in inhibiting irrelevant information and maintaining task-relevant information, which aligns with the cognitive control deficits commonly observed in depression (Berman et al., 2011). This difference in neural response patterns between anxious and depressed individuals highlights the distinct neural mechanisms underlying anxiety and depression (Foland-Ross and Gotlib, 2012; Davidson, 2002), with anxiety primarily affecting cognitive control and emotional processing through theta oscillations and depression impacting inhibitory control and information filtering through alpha oscillations (Canen and Brooker, 2017; Mizuki et al., 1997; Horvath et al., 2015; Bland and Oddie, 2001; Kaiser et al., 2018; Possel et al., 2008).

The observed correlations between neural indicators and behavioral performance provide valuable insights into the underlying mechanisms of emotional WM in anxious and depressed individuals. From a transdiagnostic perspective (Brand, 2015; Cuijpers et al., 2023), assessing anxiety and depression as continuous variables offers certain advantages over simple binary classification. For instance, in educational practice, students experiencing subclinical emotional issues (Albaugh et al., 2017) may not meet clinical diagnostic criteria but exhibit tendencies toward depression and anxiety (a group often overlooked until symptoms worsen to meet clinical criteria). Using continuous variables allows for a more effective assessment of this population. Additionally, this approach controls for comorbidities more effectively, enabling more targeted management of symptoms related to depression and anxiety.

The use of EEG as a non-invasive and cost-effective tool for assessing neural activity offers a promising approach for early detection and monitoring of anxiety and depression. The distinct electrophysiological patterns differentiating anxiety and depression symptoms during emotional WM tasks may represent quantifiable physiological features associated with subclinical emotional problems. The characterization of electrophysiological patterns associated with emotional processing differences may contribute to a more nuanced understanding of anxiety and depression manifestations, potentially guiding future research on targeted interventions. For instance, interventions aimed at enhancing cognitive flexibility and emotional regulation could be tailored based on an individual’s neural and behavioral profile, thereby improving treatment outcomes.

Despite the significant findings, several limitations should be acknowledged. First, the sample size was relatively small, which may limit the generalizability of the results. Future studies with larger and more diverse samples are needed to validate and extend these findings. Additionally, the study focused on a specific population of university students, which may not be representative of other age groups or individuals with clinical diagnoses of anxiety and depression (Foland-Ross and Gotlib, 2012; Rawlings, 2017; Divers et al., 2022). Another limitation is the reliance on self-report measures of anxiety and depression, which may be subject to response biases. Future research should incorporate objective measures, such as clinical interviews or physiological assessments, to provide a more accurate assessment of emotional states. Finally, the study focused primarily on negative emotional stimuli. While this is relevant for understanding the impact of anxiety and depression, future research should also examine the effects of positive emotional stimuli and how they interact with different cognitive loads and individual differences in emotional states.

Overall, the present study advance our understanding of electrophysiological and behavioral patterns during emotional WM processing in relation to anxiety and depression. The findings underscore the importance of considering individual differences in emotional states when examining cognitive functions and highlight how task-sensitive EEG signatures may enhance our capacity to characterize individual variability in subclinical symptom dimensions. By advancing our understanding of the complex interplay between emotion and cognition, this research contributes to the development of more effective and personalized interventions for mental health.

## Data Availability

The datasets presented in this study can be found in online repositories. The names of the repository/repositories and accession number(s) can be found in the article/supplementary material.

## Footnotes

^1^ https://osf.io/fb6xj/
